# Trends in Leishmaniasis: A 32-Year Review in an Endemic Area in the South of Madrid Region

**DOI:** 10.3390/pathogens15020127

**Published:** 2026-01-24

**Authors:** Víctor Antón-Berenguer, Óscar Manuel Muñoz Clemente, Beatriz López Quintana, Belén Martínez Mondéjar, Sara Moreno-García, Montserrat Chao Crecente, José Miguel Rubio Muñoz, Francisco Jesús Merino Fernández, Carmen Chicharro Gonzalo, Emilia García Díez, Francisco Javier Nieto Martínez, María Delmans Flores-Chávez

**Affiliations:** 1 Department of Microbiology and Parasitology, Severo Ochoa University Hospital, Av. de Orellana, s/n, 28914 Leganés, Madrid, Spain; 2 Department of Preventive Medicine, Severo Ochoa University Hospital, 28914 Leganés, Madrid, Spain; 3 Faculty of Medicine, Alfonso X El Sabio University, Av. de Orellana, s/n, 28911 Leganés, Madrid, Spain; 4 Department of Pathology, Severo Ochoa University Hospital, 28911 Leganés, Madrid, Spain; 5 Reference and Research Laboratory for Parasitology, National Centre for Microbiology, Instituto de Salud Carlos III (ISCIII), Ctra. Majadahonda-Pozuelo Km. 2, 28220 Majadahonda, Madrid, Spain; 6 Biomedical Research Centre for Infectious Diseases Network (CIBERINFEC-ISCIII), Ctra. Majadahonda-Pozuelo Km. 2, 28220 Majadahonda, Madrid, Spain; 7 World Health Organization (WHO) Collaborating Centre for Leishmaniasis, National Centre for Microbiology, ISCIII, Ctra. Majadahonda-Pozuelo Km. 2, 28220 Majadahonda, Madrid, Spain

**Keywords:** *Leishmania*, visceral leishmaniasis, cutaneous leishmaniasis, Spain, outbreak, underreporting, immunosuppression, epidemiological trends

## Abstract

In Spain, *Leishmania infantum* causes both cutaneous (CL) and visceral leishmaniasis (VL). This study aimed to analyse trends in the clinical presentation, diagnosis, management, and epidemiology of leishmaniasis at Severo Ochoa University Hospital in Leganés, an endemic area in Southern Madrid affected by Europe’s largest outbreak (2009–2015). A retrospective study was conducted, including all confirmed cases from January 1992 to December 2024, using clinical records. Cases were stratified into pre-outbreak, outbreak, and post-outbreak periods. A total of 151 cases were identified, including 129 VL, 21 CL, and 1 simultaneous VL/CL. VL predominated among adults during the HIV epidemic, later shifting to elderly and non-HIV immunosuppressed patients, while paediatric cases remained stable. Diagnostic methods evolved from bone marrow microscopy, culture, and IFAT to molecular and chemiluminescence assays. VL treatment also evolved, with amphotericin B gradually replacing meglumine antimoniate as first-line VL treatment. Most patients required hospitalisation, with 8.5% mortality, mainly among immunocompromised or elderly individuals. A persistent concentration of cases near recently urbanised areas adjacent to the parks of Polvoranca and Bosquesur was observed. Despite advances in diagnosis and therapy, endemic transmission and underreporting continue, highlighting the need for ongoing surveillance and preventive measures. Hospital record review proved useful for monitoring compliance with mandatory VL notification, though its applicability to cutaneous cases remains limited.

## 1. Introduction

Leishmaniasis is a vector-borne parasitic disease caused by the genus *Leishmania* protozoa and transmitted by sandflies of the genus *Phlebotomus* (Africa, Asia and Europe) or *Lutzomyia* (America). This disease has three main clinical forms: cutaneous leishmaniasis (CL), visceral leishmaniasis (VL), and mucocutaneous leishmaniasis (MCL). Leishmaniasis constitutes a serious global threat, remaining one of the neglected tropical diseases on the WHO list [1].

In many countries in the Mediterranean basin, including Italy, Greece, France, Portugal, and Spain, *Leishmania infantum* is endemic [2,3]. In eastern areas of Europe, isolated cases of *L. tropica*, *L. major* or *L. donovani sensu stricto* are also reported [4,5,6].

In Spain, autochthonous leishmaniasis is caused by *Leishmania infantum* via zoonotic transmission, with dogs being the main domestic reservoir. However, many other animals can also act as reservoirs, including foxes, rodents, domestic cats or hares [7]. According to the last epidemiological surveillance report (2023), 394 leishmaniasis cases were recorded in Spain, with an incidence of 0.80 cases/100,000 inhabitants. CL accounted for 47.8% of all cases, while VL made up 52.2% of cases [8].

Although the Mediterranean coast has traditionally been the area with the highest incidence of leishmaniasis in Spain, between 2009 and 2015, the South of Madrid Region experienced one of the largest leishmaniasis outbreaks in Europe, where rabbits and hares were the reservoirs [9,10].

Therefore, this study aimed to analyse trends in diagnostic testing, clinical presentation, management, and epidemiology of leishmaniasis diagnosed and treated at the Severo Ochoa University Hospital (SOUH) over thirty-two years, differentiating three different time periods: before, during, and after the leishmaniasis outbreak where the hospital directly evolved.

## 2. Materials and Methods

### 2.1. Study Design and Setting

This is a retrospective study conducted at SOUH, with a capacity of 386 beds. This hospital provided services to two municipalities in the South of Madrid Region (Spain): Leganés (194,084 inhabitants) and Fuenlabrada (194,866 inhabitants) until 2004, when Fuenlabrada hospital opened. After that, SOUH remained the reference hospital only for the municipality of Leganés.

All patients diagnosed with leishmaniasis from January 1992 to December 2024 were included in the study.

The case definition of leishmaniasis was established based on the patient’s clinical signs, exposure factors, and microbiological and/or histological findings. In recurrent leishmaniasis, only the first episode for each patient was considered.

### 2.2. Diagnostic Procedures

**Figure 1:**
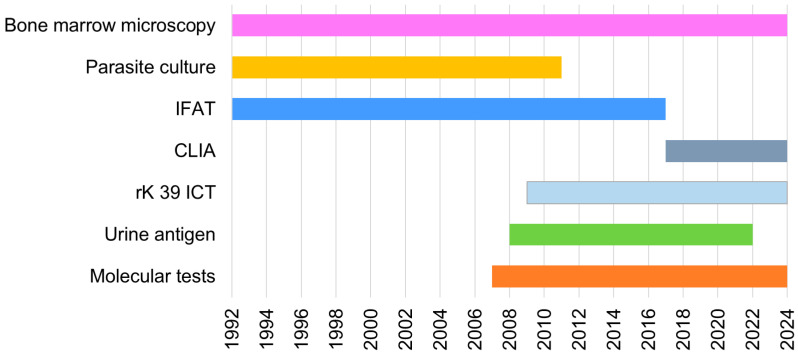
Diagnostic techniques used during the study period. Techniques were applied according to their availability. IFAT, indirect immunofluorescence antibody test; CLIA, chemiluminescence immunoassay; ICT, immunochromatographic test.

Clinical suspicion of leishmaniasis was confirmed through parasite detection or the presence of specific antibodies against it, using different tests based on their availability (Figure 1) and medical discretion. Not all tests were performed on every patient. Parasitological tests included bone marrow or biopsy microscopic examination after Giemsa or Haematoxylin–Eosin staining, culture in Novy–MacNeal–Nicolle medium provided by the National Centre for Microbiology, Instituto de Salud Carlos III (CNM-ISCIII) [11], and urine antigen detection (Katex, Kalon Biological Ltd., Guildford, United Kingdom). For molecular detection, bone marrow, blood, and/or biopsy samples were analysed at the CNM-ISCIII, using nested PCR (nPCR) or real-time PCR (qPCR) targeting the small subunit *18S rRNA* gene [12]. Anti-*Leishmania* antibodies were detected by indirect immunofluorescent antibody test (IFAT) (*Leishmania*-Spot IF, Biomerieux, Marcy-l’Étoile, France) or indirect chemiluminescent assay (CLIA) (Virclia^®^ IgG + IgM, Vircell, Granada, Spain). In cases of uncertain serological results, the rk39 immunochromatographic test (Kalazar Detect™, InBios, Seattle, USA) was employed for confirmation at CNM-ISCIII.

### 2.3. Treatment

Pharmacological treatment for leishmaniasis followed the therapeutic guidelines available at the time.

### 2.4. Variables

Data were obtained from the clinical records of the patients as well as the Microbiology, Preventive Medicine and Pathology Departments’ databases for all those patients who met the leishmaniasis case definition.

For all patients, the collected data included the following: date, age, sex, country of birth, length of residence in Spain, place of residence at the time of infection, diagnostic techniques, infection location (visceral or cutaneous), immunocompromising conditions (such as HIV, oncological/haematological conditions, diabetes, or patients receiving immunosuppressive treatments), hospital admission, length of stay, symptoms, coinfections, treatment employed, outcome, and analytical data.

VL patients were classified by immune status as follows: (i) immunocompetent (IC/VL), (ii) HIV-coinfected patients (HIV/VL), and (iii) immunosuppressed for reasons other than HIV (IS non-HIV/VL). Age groups were defined according to WHO standards as paediatric (≤15 years old), adult (16–64 years old) or elderly (≥65 years old). Considering the outbreak, cases were stratified by diagnosis date into three periods: pre-outbreak (1992–2008), outbreak peak (2009–2015), and post-outbreak (2016–2024).

### 2.5. Data Analysis and Statistical Methods

Statistical analysis was performed using “R”. Categorical variables were compared using the Chi-square test or Fisher’s exact test. To describe the precision of estimated proportions, 95% confidence intervals (95% CI) were calculated using the Wilson score method with continuity correction. Continuous variables were assessed for normality with the Shapiro–Wilk test; if normally distributed, variances were evaluated with Levene’s test and comparisons made using ANOVA. When normality or equal variances were not met, the Kruskal–Wallis test was applied. Statistical significance was defined as *p* < 0.05.

Patients without available biochemical or haematological data were excluded from analyses of these variables. For coinfections, symptoms, or laboratory abnormalities, patients could be counted in more than one category.

Incidence rates per 100,000 inhabitants were calculated using case counts and population data from the Spanish National Statistics Institute. Geographic distribution maps were created based on patients’ residence at the time of diagnosis using Google Maps.

### 2.6. Ethics

This study was approved by the Clinical Research Ethics Committee of SOUH (C.E.I.m) under code: A-1624-2024.

## 3. Results

Between 1992 and 2024, 151 patients were diagnosed with leishmaniasis, 129 (85.4%, 95% CI: 78.9–89.7) with VL, 21 (13.9%, 95% CI: 9.2–20.4) with CL, and 1 patient (0.7%, 95% CI: 0.1–3.7) presented with both clinical forms simultaneously. Among VL cases, 13 patients (10.1%, 95% CI: 6.1–16.2) exhibited uncommon clinical manifestations, with involvement of diverse histologic sites including kidney (n = 3), bronchi (n = 2), stomach (n = 2), oesophagus, vulva, rectum, vocal cords, liver or lymph node involvement. Among these thirteen patients with unusual clinical manifestations, five were HIV/VL, and four had other causes of immunosuppression, including two with haemato-oncologic conditions, one receiving methotrexate therapy, and one elderly diabetic patient. The remaining four patients did not have a formally diagnosed immunosuppressive condition but were all elderly.

Clinical suspicion of leishmaniasis was confirmed through microbiological or histological techniques in 149 patients (Table 1). In two patients with CL, no samples were sent to the microbiology or pathology departments, and the diagnosis was established based solely on the morphological assessment of the lesion by the dermatologist.

Serological tests were positive in most VL cases; however, negative serological results were observed in eight patients (seven immunocompromised and one immunocompetent). Among the seven immunocompromised patients with negative serology, one presented an unusual clinical manifestation, while the remaining six were HIV/VL. Of these patients, three were diagnosed by bone marrow microscopy, three by bone marrow culture for *Leishmania*, and the patient with unusual manifestations by biopsy histology and PCR.

Among CL cases, most were adults (n = 11), followed by elderly patients (n = 9) and one 3-year-old child. All the CL patients but two were immunocompetent, these two being non-HIV immunosuppressed. Arms were the most frequent lesion location (six cases), followed by the head (five cases) and legs (three cases). One patient presented with simultaneous lesions on the arm and leg, and another patient had simultaneous ear, thorax and arm lesions. In five cases, the lesion location was not documented. For treatment, 47.6% (10/21, 95% CI: 28.9–66.9) of them received intralesional pentavalent antimonials, and one received systemic amphotericin B treatment due to the presence of multiple lesions. In the remaining 10 patients, specific leishmaniasis treatment was not recorded.

Regarding VL patients (Table 2), 99 (76.2%, 95% CI: 68.6–82.6) of them were males and 31 (23.8%, 95% CI: 17.4–31.4) females. The confidence intervals corresponding to Table 2 are provided in Supplementary Table S1. Most of the patients, 114 (87.7%, 95% CI: 81.5–92.0), were from Spain, and 16 (12.3%, 95% CI: 7.9–18.5) were Spain-established foreigners, with a mean time living in our country of 6.7 years (range 0.5–10 years). The rates of hepatomegaly and splenomegaly were higher in paediatric and adult patients than in elderly ones (*p* < 0.05). Children presented lower rates of leukopenia, thrombopenia, and pancytopenia (*p* < 0.05), with no significant differences in anaemia (*p* = 0.08). Similarly, IS non-HIV/VL patients had lower rates of hepatomegaly and splenomegaly, whereas HIV/VL patients exhibited higher frequencies of leukopenia, pancytopenia, coinfections, and more frequent recurrences.

For management, only four patients were not hospitalised; this was one child and three of the thirteen cases with uncommon manifestations. The remaining 126 (96.9%, 95% CI: 92.7–98.7) patients required hospitalisation, with a median hospitalisation time of 14 days (range 1–87 days). Ten patients (7.7%, 95% CI: 4.3–13.4) required intensive care unit admission: one child, three elderly patients, four immunosuppressed patients (two HIV, one a haematologic patient and another with an immunosuppressive corticosteroid treatment), and two immunocompetent adult patients.

Stratification of VL cases considering the outbreak revealed notable shifts in patient profiles (Table 3). A diminution of HIV/VL patients between the first and second periods was noticed, while the prevalence of IS non-HIV/VL increased. In the same way, the adult population also decreased between the first and second periods, with an increase in the elderly population.

**Figure 2:**
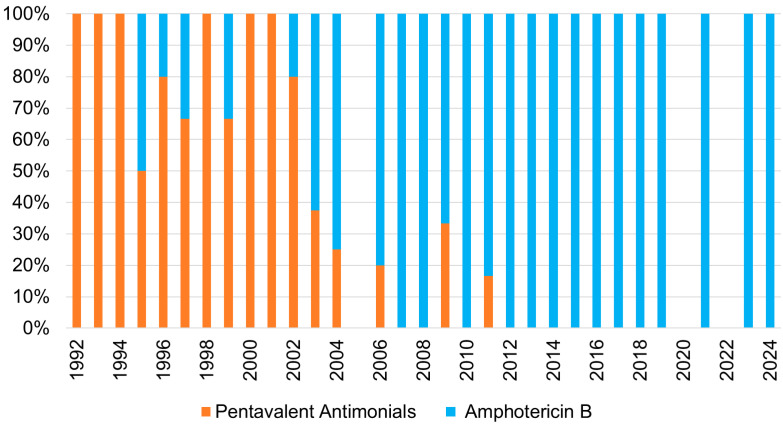
Treatment employed for the management of VL patients over time. Pentavalent antimonials were progressively replaced by amphotericin B, which became the predominant therapy from 2003 onwards.

Regarding VL treatment, pentavalent antimonials were the most used drug (47/130) between 1992 and 2002 (Figure 2), with their administration being progressively discontinued and replaced by Amphotericin B, which became the most employed drug (76/130), with both having similar hospitalisation times (*p* = 0.3). Among patients treated with pentavalent antimonials, complete cure was achieved in thirty-six cases (76.6%, 95% CI: 63.0–86.3); nine patients (19.1%, 95% CI: 10.1–33.1) experienced at least one recurrence, and two (4.3%, 95% CI: 1.2–14.6) died. Among those treated with Amphotericin B, 55 patients (72.3%, 95% CI: 61.0–81.3) achieved complete cure, 12 (15.8%, 95% CI: 9.1–26.3) experienced recurrences, and 9 (11.9%, 95% CI: 6.3–21.3) died. No statistically significant differences in outcomes were observed between the two treatment groups. In the rest of the patients, one oncologic–haematologic patient diagnosed with bronchial leishmaniasis was treated with miltefosine as monotherapy and achieved complete cure. In four cases, the treatment employed was unknown. In two cases, specific antileishmanial treatment was not administered; one case was a paediatric patient with prolonged fever, diagnosed through urinary antigen and blood-PCR who was initially misdiagnosed with urinary tract infection and empirically treated with amoxicillin/clavulanate, and one case of ganglionic leishmaniasis causing lymphadenitis which self-healed.

Concerning the outcomes of VL patients, 97 cases (74.6%, 95% CI: 66.8–81.3) achieved complete cure, 22 (16.9%, 95% CI: 11.3–24.6) experienced at least one recurrence, and 11 (8.5%, 95% CI: 4.8–15.0) died. Fatal cases were associated with concomitant infectious or chronic diseases: four patients were coinfected with HIV, one was receiving corticosteroid immunosuppressive therapy, one had an oncological condition, and five were elderly, two of whom also having underlying haematological disorders.

**Figure 3:**
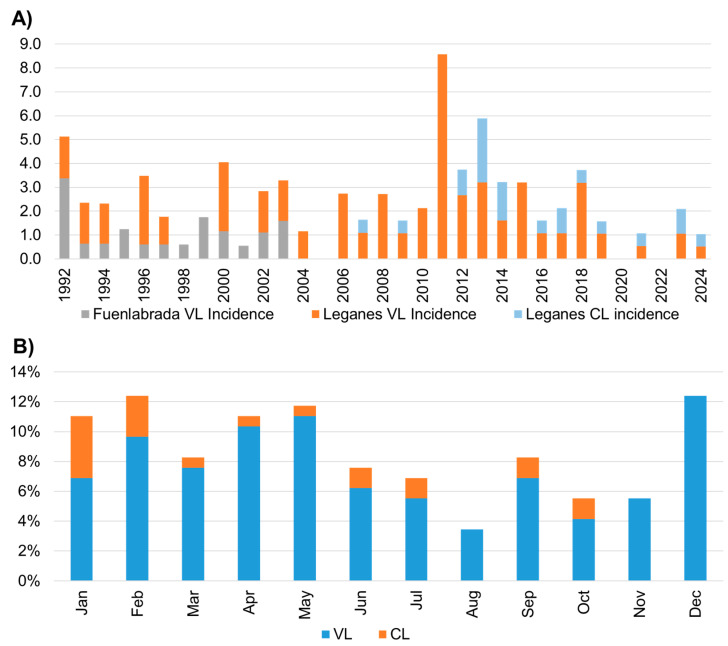
Temporal distribution of leishmaniasis cases in the SOUH area. (**A**) Annual incidence of VL and CL between 1992 and 2024, with a peak in 2011. (**B**) Monthly distribution showing seasonal peaks from January to May and in December.

The total incidence of leishmaniasis in the SOUH area was 1.9 cases/100,000 inhabitants, 1.6 cases/100,000 inhabitants for VL, and 0.3 cases/100,000 inhabitants for CL. Incidence fluctuated over the study period, reaching an important peak of almost 9 cases/100,000 inhabitants in 2011, and a decrease in positivity rate in the period from June to November throughout the year (Figure 3).

**Figure 4:**
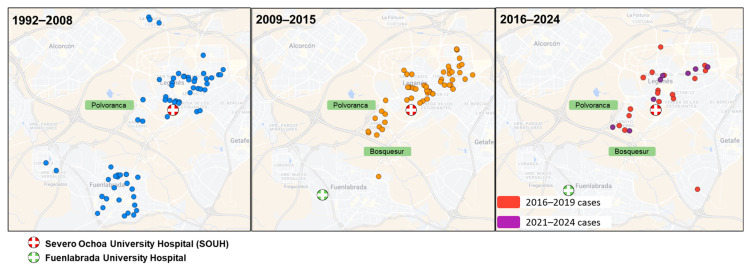
Geographical distribution of leishmaniasis cases in the SOUH area across three periods. An apparent concentration of cases was observed around Polvoranca park during the outbreak period, persisting in the subsequent years. SOUH served as the reference hospital for Leganés and Fuenlabrada until 2004; since 2004, leishmaniasis patients from Fuenlabrada have been managed at Fuenlabrada Hospital. Dot colours indicate different study periods, as shown in the legend.

When assessing the spatial distribution of leishmaniasis cases in Leganés (Figure 4), an apparent concentration of cases was observed in areas surrounding Polvoranca park during the outbreak period. This pattern persisted in the post-outbreak period.

## 4. Discussion

This study reviews leishmaniasis in an endemic area in the South of the Madrid Region, one of the areas affected by the largest outbreak reported in Spain and the Mediterranean region, focusing on changes in diagnosis, clinical manifestations, management, and epidemiological trends.

Clinically, VL predominated over CL, 85.4% vs. 13.9%, in contrast with the East part of Madrid, where, from 1987 to 2016, these proportions were 62.3% vs. 37.7% [13]. In our review, CL increased from the outbreak period (Table 1, Figure 3A). This data can likely be attributed to the educational effect of the outbreak, which heightened clinicians’ awareness, prompting them to consider CL when dealing with patients presenting cutaneous lesions. Before the outbreak, the lack of experience among primary care doctors and the self-limiting nature may have contributed to CL underrepresentation. Nevertheless, between 2016 and 2024, the CL rate of our study was 36%, still far from the data of nationwide reports, 41.2% (2016–2017) [14] and 47.8% (2023) [8], highlighting the need for greater efforts in the improvement of CL diagnosis and its proper notification. No cases of MCL nor post-kala-azar dermal leishmaniasis were reported in this study although these are uncommon; some cases of *L. infantum* causing these manifestations have been reported in other areas of Madrid [15,16].

In accordance with WHO recommendations, diagnosis of leishmaniasis at SOUH is made by combining clinical manifestations with lab tests. IFAT was the primary serological technique used to confirm VL clinical suspicion in combination with bone marrow microscopy or parasite culture (Figure 1, Table 1). From 2007, the introduction of PCR represented an advancement, as this test allows the use of less-invasive samples like blood [12]. However, PCR on bone marrow samples (Table 1) continued to demonstrate higher sensitivity as highlighted by Cruz et al. [17]. Additionally, this sample allows the differential diagnosis of haematologic conditions.

Similarly, for VL diagnosis, rapid diagnostic tests were introduced, including urine antigenic detection and rk39 ICT. Unfortunately, the sensitivity of antigenic detection was only around 50% (Table 1), as previously reported [18], probably due to its sustainability not being possible; this test was therefore discontinued despite the sampling being less invasive.

Despite IFAT’s usefulness, it was discontinued from 2017, and replaced with CLIA, which, although yielding positive results in all VL patients tested in our series, has limited data on specificity, highlighting the need for highly specific confirmation methods, such as rk39 [19].

The progressive introduction of molecular and less-invasive diagnostic techniques over the study period may also have influenced case detection and the apparent epidemiological patterns observed. The availability of PCR-based assays and blood-based molecular testing facilitated the diagnosis of VL in elderly patients and in individuals with comorbidities or immunosuppressive treatments, for whom invasive procedures such as bone marrow aspiration may have been delayed or avoided. However, as molecular testing was often performed in reference laboratories, access to PCR may also have influenced the timing of diagnostic confirmation in some cases.

Although molecular methods offer high sensitivity, particularly for low-parasite-burden infections, serology has remained a widely available and rapid diagnostic tool in our setting. The combined use of serological and molecular approaches was especially valuable in severely immunocompromised patients, in whom serology may be negative, as observed in seven cases in our series (Table 1).

Similarly, for CL, molecular testing enabled the detection of cases with low parasite burdens that might otherwise have remained undiagnosed using conventional microscopy or culture. The introduction of PCR in 2007 coincided with the identification of the first CL cases in our cohort (Figure 1, Table 1), suggesting that part of the increase in CL diagnoses reflects improved diagnostic sensitivity rather than a true rise in incidence.

Taken together, these diagnostic advances likely reduced diagnostic delay in some patients and expanded the spectrum of detectable disease and should therefore be considered when interpreting the temporal and demographic trends observed in this 32-year series.

No imported cases of leishmaniasis were reported in this study. At the time of diagnosis, all foreign patients had been living in Spain for more than six months, exceeding the incubation period for leishmaniasis and ruling out the possibility of an imported case.

As typically reported, children remain one of the most vulnerable populations at risk of developing VL [20]. This group presented similar male/female infection rates (54.2% and 47.4%, respectively), consistent with other studies [21,22]. Conversely, the rest of the age groups showed a clear male predominance, which is associated with both biological and socio-cultural factors [23]. With the spread of HIV infection, HIV-related immunocompromised patients became the predominant VL-affected population until the introduction of effective antiviral treatments [24]. Those patients demonstrated higher rates of leukopenia and pancytopenia, as noticed by Ramos et al. [25].

In recent decades, both population ageing, with its associated immune system decline, and the increasing use of immunosuppressive treatments have given rise to new at-risk groups for VL infection, with these patients showing lower rates of hepatomegaly and splenomegaly [26,27].

In Spain, leishmaniasis has been a notifiable disease since 1982, with reporting becoming the responsibility of regional authorities in 1995. Due to the increase in cases during the outbreak (Figure 3A) [9], national mandatory reporting was reinstated in 2015. Nevertheless, underreporting remains a persistent issue. Our findings show that hospital record reviews allow evaluation of reporting compliance and can complement surveillance bulletins by providing more up-to-date information. While reporting is generally adequate for VL, CL continues to be underreported, as during the outbreak, the Madrid Region surveillance system recorded 24 CL cases, whereas only 9 were documented in patients’ medical histories at SOUH [11].

No cases were detected in 2020, likely due to the restrictions imposed during the SARS-CoV-2 pandemic. That year, only 26 cases were reported in Madrid [8]. In subsequent years (Figure 3A), new cases re-emerged as in all the country (46 cases in Madrid in 2021) [8].

The seasonal distribution of cases showed a marked decline between June and November, coinciding with phlebotomines’ most active periods in this area (Figure 3B) [11,28]. This pattern, reflecting the long incubation period for leishmaniasis, was already observed in another study [28].

The geographical distribution of cases showed a higher concentration in newly urbanised areas adjacent to Polvoranca and Bosquesur, two locations that underwent major environmental and infrastructural changes around the time of the outbreak (Figure 4). Similar spatial patterns have been described in other settings, where human-driven landscape modifications have been associated with changes in leishmaniasis transmission dynamics [29,30].

Beyond the increase in incidence observed during 2009–2015 (Figure 3), this long-term series suggests that the epidemiological impact of a large outbreak may extend well beyond its apparent end. In our setting, cases remained concentrated in the same areas in the subsequent years, supporting the concept that outbreaks can function as inflexion points that shape local transmission patterns over prolonged periods rather than representing strictly transient events. From a control perspective, these observations argue for sustained post-outbreak surveillance and prevention efforts, instead of assuming an automatic return to pre-outbreak conditions.

The post-outbreak phase therefore merits particular consideration. Although overall incidence declined after 2015 and approached values similar to those observed before the outbreak (Figure 3), this does not necessarily indicate a full return to the previous transmission baseline. Instead, our data are compatible with the establishment of a new endemic equilibrium, in which transmission persists at lower intensity but remains spatially focused in the same areas affected during the outbreak. In our series, the sustained proportion of cases in areas surrounding Polvoranca and Bosquesur after 2015 supports this interpretation (Figure 4).

Although our analysis is descriptive and no formal spatial clustering tests were performed, the continued concentration of cases in these locations suggests that post-outbreak control measures may reduce overall incidence without fully eliminating local transmission foci. This persistence is in line with previous investigations of the South Madrid outbreak, which documented the continued presence of infected animal reservoirs, particularly rabbits and hares, and active phlebotomine vectors despite declining infection rates after the implementation of control measures [31].

A second transferable lesson from this 32-year dataset is the dynamic nature of risk groups for VL in Europe. During the earlier period, HIV/VL accounted for a substantial proportion of cases, consistent with the epidemiological context of the HIV epidemic. Over time, and particularly after the widespread availability of effective antiretroviral therapy, this profile shifted towards an increasing representation of elderly patients and individuals receiving immunosuppressive therapies for non-HIV conditions. This transition has direct clinical relevance, as it broadens the spectrum of patients in whom leishmaniasis should be considered and highlights the need for diagnostic vigilance beyond traditional high-risk profiles.

These findings also have implications for surveillance and clinical practice in other endemic European regions. First, long-term hospital-based retrospective reviews may complement routine notification systems by providing granular insight into case profiles and potential underreporting patterns. Second, clinicians in endemic areas should maintain awareness of leishmaniasis not only during outbreaks, but also in the post-outbreak period, particularly among elderly and immunosuppressed patients. Finally, our data support the need to reinforce notification and reporting pathways, especially for CL, in order to reduce underreporting and to improve early detection of epidemiological changes that may herald new outbreaks.

The main limitation of our study lies in its retrospective design. As not all diagnostic tests were performed on every patient, the sensitivity and specificity values for the different tests could not be calculated. Nevertheless, all the techniques employed in this study have been previously validated, demonstrating their reliability [12].

In summary, leishmaniasis has undergone substantial changes over the past decades. The profile of patients with VL has shifted from a predominance of HIV/VL individuals to an increasing representation of elderly patients and those receiving non-HIV immunosuppressive therapies. In parallel, clinical management has evolved, with safer treatments and a transition from traditional diagnostic methods to molecular, rapid, and highly sensitive techniques.

Beyond these clinical advances, this long-term experience highlights that the epidemiological impact of a large outbreak may extend well beyond the outbreak period, reinforcing the need for sustained surveillance and clinical awareness in endemic European settings. Despite improvements in diagnosis and care, underreporting—particularly of CL—remains a persistent challenge that warrants continued attention.

## Figures and Tables

**Table 1:** Diagnostic tools employed for leishmaniasis confirmation.

	VL n = 130			CL n = 21 ^a^		
	1992–2008 n = 70	2009–2015 n = 44	2016–2024 n = 16	1992–2008 n = 1	2009–2015 n = 11	2016–2024 n = 9
Bone marrow microscopy (42/69)	31/47	11/19	0/3			
Parasite culture (19/39)	14/27	5/12				
IFAT (97/107)	56/64	38/40	3/3			
CLIA (13/13)			13/13			
rk39 ICT (11/12)		7/8	4/4			
Urine antigen (25/48)	2/3	18/35	5/10			
Bone marrow PCR (30/35)	2/5	21/22	7/8			
Blood PCR (24/31)	2/3	15/21	7/7			
Organ biopsy microscopy (13/13)	3/3	7/7	3/3			
Organ biopsy PCR (3/3)		3/3				
Skin biopsy microscopy (7/8) ^b^	1/1				1/1	5/6
Skin biopsy PCR (15/15)				1/1	8/8	6/6

(n/n), positive tests/performed tests; IFAT, immunofluorescence antibody test; CLIA, chemiluminescence antibody assay; ICT, immunochromatographic test. ^a^ Two patients were diagnosed based on the morphological assessment of the lesion. ^b^ One patient presented simultaneous VL and CL. VL was confirmed by IFAT and bone marrow microscopy, and CL by skin biopsy microscopy.

**Table 2:** Description of characteristics of VL patients according to their age group and immune status.

	Paediatrics	Adults	Elder	*p*	IC	HIV	IS Non-HIV	*p*
	n = 24	n = 83	n = 23		n = 72	n = 38	n = 20	
**Age**								
Median age (IQR)	1 (0.9)	36.2 (15.3)	74.1 (6.8)	-	31.4 (53.9)	34 (7.7)	64.4 (24.6)	**0.005**
**Gender**								
Female n (%)	11 (45.8)	16 (19.3)	4 (17.4)	**0.01**	19 (26.4)	6 (15.8)	6 (30)	0.4
Male n (%)	13 (54.2)	67 (80.7)	19 (82.6)	**0.01**	53 (73.6)	32 (84.2)	14 (70)	0.4
**Clinical manifestations**								
Fever n (%)	20 (83.3)	73 (87.9)	13 (56.5)	**0.003**	61 (84.1)	32 (84.2)	13 (65)	0.1
Hepatomegaly n (%)	19 (79.2)	50 (60.2)	9 (39.1)	**0.01**	42 (58.3)	29 (76.3)	7 (40)	**0.02**
Splenomegaly n (%)	22 (91.7)	63 (75.9)	10 (43.5)	**0.005**	61 (84.7)	28 (73.7)	6 (30)	**0.005**
Gastrointestinal symptoms n (%)	5 (20.8)	19 (22.8)	0 (0)	**0.04**	12 (16.7)	10 (26.3)	2 (10)	0.3
Respiratory symptoms n (%)	3 (12.5)	18 (21.7)	4 (17.4)	0.6	14 (19.4)	7 (18.4)	4 (25)	0.4
General symptoms ^a^ n (%)	5 (20.8)	36 (43.4)	7 (30.4)	0.2	23 (31.9)	16 (42.1)	9 (45)	0.4
**Analytical alterations**								
Anaemia ^b^ n (%)	22 (91.7)	63 (75.9)	15 (65.2)	0.08	56 (77.8)	29 (76.3)	15 (75)	0.1
Thrombopenia ^c^ n (%)	9 (37.5)	60 (72.3)	12 (52.2)	**0.007**	41 (56.9)	28 (73.7)	12 (60)	0.9
Leukopenia n (%)	7 (29.2)	65 (78.3)	15 (65.5)	**0.005**	44 (61.1)	31 (81.6)	12 (60)	**0.03**
Pancytopenia n (%)	3 (12.5)	50 (60.2)	9 (39.1)	**0.005**	31 (43.1)	26 (68.4)	5 (25)	**0.005**
Icteric ^d^ n (%)	0 (0)	8 (9.6)	1 (4.3)	0.2	4 (5.6)	5 (13.2)	0 (0)	0.1
GPT > 45 U/L n (%)	9 (37.5)	28 (33.7)	7 (30.4)	0.8	30 (41.7)	8 (21.1)	6 (30)	0.08
GOT > 34 U/L n (%)	17 (70.8)	42 (50.6)	9 (39.1)	0.06	41 (56.9)	19 (50)	8 (40)	0.4
CRP mean (IQR)	79.7 (58.5)	104.3 (141)	77.5 (125)	0.2	111.2 (106)	50.8 (42.4)	72.7 (100.1)	**0.005**
**Coinfections**								
N	2 (8.3)	39 (47)	5 (21.7)	**0.005**	12 (16.7)	29 (76.3)	5 (25)	**0.005**
Bacteria ^e^ n (%)	0 (0)	18 (21.7)	3 (13)	**0.03**	6 (8.3)	12 (31.5)	3 (15)	**0.005**
HIV n (%)	0 (0)	38 (45.8)	0 (0)	**<0.005**	0 (0)	38 (100)	0 (0)	
Virus (non-HIV) ^f^ n (%)	1 (4.2)	25 (30.1)	2 (8.7)	**0.002**	5 (6.9)	22 (57.9)	1 (5)	**0.005**
Fungi ^g^ n (%)	1 (4.2)	15 (18.1)	1 (4.3)	**0.04**	2 (2.8)	13 (34.2)	2 (10)	**0.005**
Parasites ^h^ n (%)	0 (0)	4 (4.8)	0 (0)	0.3	0 (0)	4 (10.5)	0 (0)	**0.007**
**Outcome**								
Curation n (%)	21 (87.5)	60 (72.3)	16 (69.6)	0.1	61 (84.7)	21 (55.2)	15 (75)	**0.006**
Recidivist n (%)	3 (12.5)	17 (20.5)	2 (8.7)	0.4	8 (11.1)	13 (34.2)	1 (5)	**0.003**
Exitus n (%)	0 (0)	6 (7.2)	5 (21.7)	**0.02**	3 (4.2)	4 (10.5)	4 (20)	0.07

^a^ General symptoms include asthenia, headache, general discomfort or shivering. ^b^ Hb < 11.6 g/dL for women. Hb < 13.2 g/dL for men. ^c^ Platelets < 130 × 10^3^/µL. ^d^ Bilirubin < 1.2 mg/dL. CRP, C-reactive protein. ^e^ *Mycobacterium tuberculosis* n = 10, *Coxiella burnetii* n = 4, *Neisseria gonorrhoeae* n = 2, *Streptococcus pneumoniae* n = 1, *Haemophilus influenzae* n = 1, *Staphylococcus aureus* n = 1, *Stenotrophomonas maltophilia* n = 1, *Treponema pallidum* n = 1, *Campylobacter jejuni* n = 1. ^f^ Hepatitis C virus n = 17, Hepatitis B virus n = 9, Hepatitis A virus n = 2, Cytomegalovirus n = 3, Herpes simplex virus I n = 1, Varicella-Zoster virus n = 1, Herpes simplex virus VIII n = 1, Rotavirus n = 1. ^g^ Oral candidiasis n = 14, *Pneumocystis jirovecii* n = 5, genital candidiasis n = 1, pulmonar aspergillosis n = 1. ^h^ Brain toxoplasmosis n = 2, *Giardia lamblia* n = 1, *Entamoeba* spp. n = 1. Values in bold indicate statistically significant *p* values.

**Table 3:** Distribution of VL cases across periods before (1992–2008), during (2009–2015), and after (2016–2024) the outbreak.

	1992–2008 n = 70	2009–2015 n = 44	2016–2024 n = 16	*p*
**Immune status**				
IC/VL n (%; 95% CI)	33 (47.1; 35.9–58.7)	27 (61.4; 46.6–74.3)	12 (75.0; 50.5–89.8)	0.2
HIV/VL n (%; 95% CI)	33 (47.1; 35.9–58.7)	4 (9.1; 3.6–21.2)	1 (6.2; 1.1–28.3)	**<0.005**
IS non-HIV/VL n (%; 95% CI)	4 (5.7; 2.2–13.8)	13 (29.5; 18.2–44.2)	3 (18.8; 6.6–43.0)	**<0.005**
**Age group**				
Paediatrics n (%; 95% CI)	9 (12.9; 6.9–22.7)	13 (29.5; 18.2–44.2)	2 (12.5; 3.5–36.0)	0.08
Adults n (%; 95% CI)	57 (81.4; 70.8–88.8)	17 (38.6; 25.7–53.4)	9 (56.2; 33.2–76.9)	**<0.005**
Elderly n (%; 95% CI)	4 (5.7; 2.2–13.8)	14 (31.8; 20.0–46.6)	5 (31.2; 14.2–55.6)	**<0.005**
**Outcome**				
Curation n (%; 95% CI)	52 (74.3; 63.0–83.1)	31 (70.5; 55.8–81.8)	14 (87.5; 64.0–96.5)	0.4
Recidivist n (%; 95% CI)	13 (18.6; 11.2–29.2)	9 (20.5; 11.2–34.5)	0 (0.0; 0.0–19.4)	0.2
Exitus n (%; 95% CI)	5 (7.1; 3.1–15.7)	4 (9.1; 3.6–21.2)	2 (12.5; 3.5–36.0)	0.8

Values in bold indicate statistically significant *p* values.

## Data Availability

The data that support the findings of this study are not publicly available due to privacy and data protection restrictions. However, the data are held by the corresponding author and may be shared upon reasonable request, subject to appropriate ethical and legal considerations.

## Supplementary Materials

The following supporting information can be downloaded at: https://www.mdpi.com/article/10.3390/pathogens15020127/s1, Table S1: Proportions and 95% confidence intervals (95% CI) for the clinical and epidemiological characteristics of leishmaniasis cases shown in Table 2.
