# Supplementary material for: Trends in Leishmaniasis: A 32-Year Review in an Endemic Area in the South of Madrid Region
Source: Pathogens. 2026 Jan 24;15(2):127. doi: 10.3390/pathogens15020127 (PMC12943515; doi:10.3390/pathogens15020127)
Supplement: Supplementary file 1 [file pathogens-15-00127-s001.zip › pathogens-4057348-supplementary.pdf]

Table S1. Proportions and 95% confidence intervals (95% CI) for the clinical and epidemiological characteristics of leishmaniasis cases shown in Table 2.

	**Paediatrics** n = 24	**Adults** n = 83	**Elderly** n = 23	*p*	**IC** n = 72	**HIV** n = 38	**IS non-HIV** n = 20	*p*
Age								
Median age (IQR)	1 (0.9)	36.2 (15.3)	74.1 (6.8)	-	31.4 (53.9)	34 (7.7)	64.4 (24.6)	***0.005***
Gender								
Female n (%; 95%CI)	11 (45.8; 27.9-64.9)	16 (19.3; 12.2-29.0)	4 (17.4; 7.0-37.1)	***0.01***	19 (26.4; 17.6–37.6)	6 (15.8; 7.5–30.2)	6 (30.0; 14.6–51.9)	*0.4*
Male n (%; 95%CI)	13 (54.2; 35.1-72.1)	67 (80.7; 71.0-87.8)	19 (82.6; 62.9-93.0)	***0.01***	53 (73.6; 62.4–82.4)	32 (84.2; 69.8–92.5)	14 (70.0; 48.1–85.4)	*0.4*
Clinical manifestations								
Fever n (%; 95%CI)	20 (83.3; 64.1-93.3)	73 (88.0; 79.2-93.3)	13 (56.5; 36.8-74.4)	***0.003***	61 (84.7; 74.2–91.4)	32 (84.2; 69.8–92.5)	13 (65.0; 43.3–82.0)	*0.1*
Hepatomegaly n (%; 95%CI)	19 (79.2; 59.5-90.8)	50 (60.2; 49.5-70.1)	9 (39.1; 22.2-59.2)	***0.01***	42 (58.3; 46.6–69.2)	29 (76.3; 60.8–87.0)	7 (40.0; 21.9–61.3)	***0.02***
Splenomegaly n (%; 95%CI)	22 (91.7; 74.2-97.7)	63 (75.9; 65.7-83.8)	10 (43.5; 25.6-63.2)	***0.005***	61 (84.7; 74.2–91.4)	28 (73.7; 58.1–85.2)	6 (30.0; 14.6–51.9)	***0.005***
Gastrointestinal symptoms n (%; 95%CI)	5 (20.8; 9.2-40.5)	19 (22.9; 15.2-33.0)	0 (0.0; 0.0-14.3)	***0.04***	12 (16.7; 9.8–27.1)	10 (26.3; 15.0–42.0)	2 (10.0; 2.8–30.1)	*0.3*
Respiratory Symptoms n (%; 95%CI)	3 (12.5; 4.3-31.0)	18 (21.7; 14.2-31.7)	4 (17.4; 7.0-37.1)	*0.6*	14 (19.4; 12.0–30.0)	7 (18.4; 9.2–33.7)	4 (20.0; 8.1–41.6)	*0.4*
General Symptoms [a] n (%; 95%CI)	5 (20.8; 9.2-40.5)	36 (43.4; 33.2-54.1)	7 (30.4; 15.6-50.9)	*0.2*	23 (31.9; 22.3–43.3)	16 (42.1; 27.0–58.8)	9 (45.0; 25.8–65.8)	*0.4*
Analytical alterations								
Anaemia [b] n (%; 95%CI)	22 (91.7; 74.2-97.7)	63 (75.9; 65.7-83.8)	15 (65.2; 44.9-81.2)	*0.08*	56 (77.8; 66.8–85.8)	29 ((76.3; 60.8–87.0)	15 (75.0; 53.1–88.8)	*0.1*
Thrombopenia [c] n (%; 95%CI)	9 (37.5; 21.2-57.3)	60 (72.3; 61.8-80.8)	12 (52.2; 33.0-70.8)	***0.007***	41 (56.9; 45.3–67.7)	28 (73.7; 58.1–85.2)	12 (60.0; 38.7–78.1)	*0.9*
Leukopenia n (%; 95%CI)	7 (29.2; 14.9-49.2)	65 (78.3; 68.3-85.8)	15 (65.2; 44.9-81.2)	***0.005***	44 (61.1; 49.4–71.7)	31 (81.6; 66.6–90.8)	12 (60.0; 38.7–78.1)	***0.03***
Pancytopenia n (%; 95%CI)	3 (12.5; 4.3-31.0)	50 (60.2; 49.5-70.1)	9 (39.1; 22.2-59.2)	***0.005***	31 (43.1; 32.1–54.9)	26 (68.4; 52.5–80.9)	5 (25.0; 10.7–47.2)	***0.005***
Icteric [d] n (%; 95%CI)	0 (0.0; 0.0-13.8)	8 (9.6; 5.0-17.9)	1 (4.3; 0.8-21.0)	*0.2*	4 (5.6; 2.2–13.4)	5 (13.2; 5.8–27.1)	0 (0.0; 0.0–16.8)	*0.1*
GPT > 45 U/L n (%; 95%CI)	9 (37.5; 21.2-57.3)	28 (33.7; 24.5-44.4)	7 (30.4; 15.6-50.9)	*0.8*	30 (41.7; 30.6–53.7)	8 (21.1; 11.1–36.6)	6 (30.0; 14.6–51.9)	*0.08*
GOT > 34U/L n (%; 95%CI)	17 (70.8; 50.8-85.1)	42 (50.6; 40.1-61.1)	9 (39.1; 22.2-59.2)	*0.06*	41 (56.9; 45.3–67.7)	19 (50.0; 34.5–65.5)	8 (40.0; 21.9–61.3)	*0.4*
CRP mean (IQR)	79.7 (58.5)	104.3 (141)	77.5 (125)	*0.2*	111.2 (106)	50.8 (42.4)	72.7 (100.1)	***0.005***
Coinfections								
N	2 (8.3; 2.3-25.8)	39 (47.0; 36.6-57.6)	5 (21.7; 9.7-41.9)	***0.005***	12 (16.7; 9.8–27.1)	29 (76.3; 60.8–87.0)	5 (25.0; 10.7–47.2)	***0.005***
Bacteria [e] n (%; 95%CI)	0 (0.0; 0.0-13.8)	18 (21.7; 14.2-31.7)	3 (13.0; 4.5-32.1)	***0.03***	6 (8.3; 3.9–17.0)	12 (31.5; 18.5–48.6)	3 (15.0; 5.2–36.0)	***0.005***
HIV n (%; 95%CI)	0 (0.0; 0.0-13.8)	38 (45.8; 35.5-56.4)	0 (0.0; 0.0-14.3)	***<0.005***	0 (0.0; 0.0–5.1)	38 (100.0; 90.8–100.0)	0 (0.0; 0.0–16.8)	-
Virus (non-HIV) [f] n (%; 95%CI)	1 (4.2; 0.7-20.2)	25 (30.1; 21.3-40.7)	2 (8.7; 2.4-26.8)	***0.002***	5 (6.9; 3.0–15.2)	22 (57.9; 41.5–72.8)	1 (5.0; 0.9–23.6)	***0.005***
Fungi [g] n (%; 95%CI)	1 (4.2; 0.7-20.2)	15 (18.1; 11.3-27.7)	1 (4.3; 0.8-21.0)	***0.04***	2 (2.8; 0.8–9.5)	13 (34.2; 20.7–50.8)	2 (10.0; 2.8–30.1)	***0.005***
Parasites [h] n (%; 95%CI)	0 (0.0; 0.0-13.8)	4 (4.8; 1.9-11.7)	0 (0.0; 0.0-14.3)	*0.3*	0 (0.0; 0.0–5.1)	4 (10.5; 4.2–24.1)	0 (0.0; 0.0–16.8)	***0.007***
Outcome								
Curation n (%; 95%CI)	21 (87.5; 69.0-95.7)	60 (72.3; 61.8-80.8)	16 (69.6; 49.1-84.4)	*0.1*	61 (84.7; 74.2–91.4)	21 (55.2; 39.0–70.4)	15 (75.0; 53.1–88.8)	***0.006***
Recidivist n (%; 95%CI)	3 (12.5; 4.3-31.0)	17 (20.5; 13.2-30.4)	2 (8.7; 2.4-26.8)	*0.4*	8 (11.1; 5.6–20.8)	13 (34.2; 20.7–50.8)	1 (5.0; 0.9–23.6)	***0.003***
Exitus n (%; 95%CI)	0 (0.0; 0.0-13.8)	6 (7.2; 3.4-14.9)	5 (21.7; 9.7-41.9)	***0.02***	3 (4.2; 1.4–11.5)	4 (10.5; 4.2–24.1)	4 (20.0; 8.1–41.6)	*0.07*
